# Renal fungal ball—two case reports and review of literature

**DOI:** 10.1259/bjrcr.20150247

**Published:** 2016-05-08

**Authors:** Devimeenal Jegannathan, Kanagasabai Ramanathan

**Affiliations:** Department of Radiology, Government Kilpauk Medical College, Dr MGR Medical University, Chennai, India

## Abstract

Fungal ball or fungal bezoar is the saprophytic colonization of a pre-formed cavity by a conglomerate of fungal mycelia without invasion of the adjacent tissue. Fungal bezoar is seen commonly in immunocompromised individuals. We describe the cross-sectional imaging characteristics of two cases of renal fungal ball, a rare clinical entity. The first case is that of a 36-year-old female with diabetes who presented with a single fungal ball that caused ballooning of the renal pelvis by coelomycetes, a rare species of fungi. The second case is that of a 45-year-old immunosuppressed male with diabetes who presented with multiple *Candida albicans* fungal balls and emphysematous pyelonephritis. Awareness about the various imaging findings of this rare clinical entity and a high index of suspicion in high-risk individuals will help in overcoming the challenges in early diagnosis and, thereby, institution of proper treatment.

## Summary

Fungal ball or fungal bezoar is the saprophytic colonization of a pre-formed cavity by a conglomerate of fungal mycelia without invasion of the adjacent tissue. Fungal bezoar is seen commonly in immunocompromised individuals. We describe the cross-sectional imaging characteristics of two cases of renal fungal ball, a rare clinical entity. The first case is that of a 36-year-old female with diabetes who presented with a single fungal ball that caused ballooning of the renal pelvis by coelomyecetes, a rare species of fungi. The second case is that of a 45-year-old immunosuppressed male with diabetes who presented with multiple *Candida albicans* fungal balls and emphysematous pyelonephritis (EPN). Awareness about the various imaging findings of this rare clinical entity and a high index of suspicion in high-risk individuals will help in overcoming the challenges in early diagnosis and, thereby, institution of proper treatment.

## Clinical presentation—case 1

A 36-year-old female patient with diabetes was admitted with complaints of increased frequency of urine for 1 week, fever, vomiting, right loin pain for 2 weeks, and nocturnal incontinence and urgency for 3 months.

The patient had a history of right renal calculus, for which extracorporeal shock wave lithotripsy and double J stenting were performed 4 years back. 10 years back, she underwent right mastectomy for carcinoma of the breast that was followed by chemotherapy.

Routine urine analysis showed 2–4 pus cells and 2–3 red blood cells. Urine culture showed species of *Enterococcus* and *Pseudomonas*, sensitive to amikacin.

### Differential diagnosis

Provisional clinical diagnosis on admission was pelviureteric junctional calculus and an associated bacterial infection.

### Imaging findings

**Figure 1.**
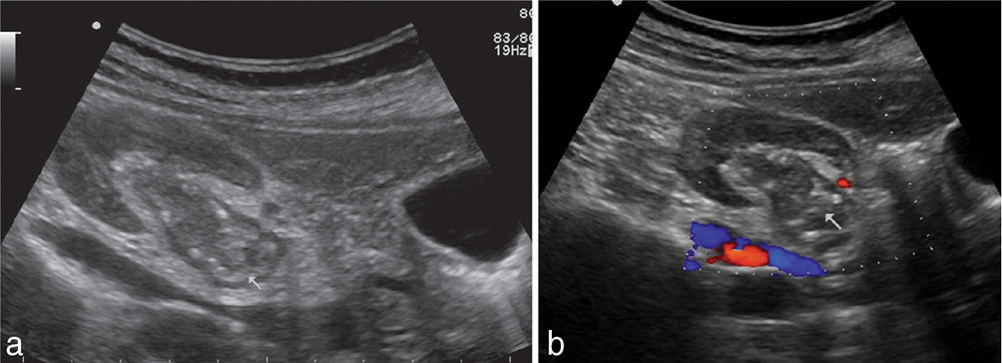
Ultrasonogram (a) and colour Doppler (b) images of the right kidney showing an isoechoic lesion (white arrows) in the renal pelvis with a peripheral rim of calcification and absence of vascularity.

The patient was referred for ultrasonogram (USG) of the abdomen, which showed a predominantly hypoechoic lesion in the dilated right renal pelvis with specks of echogenic foci in the periphery (Figure 1a). It did not show any colour flow (Figure 1b) on Doppler study.

**Figure 2.**
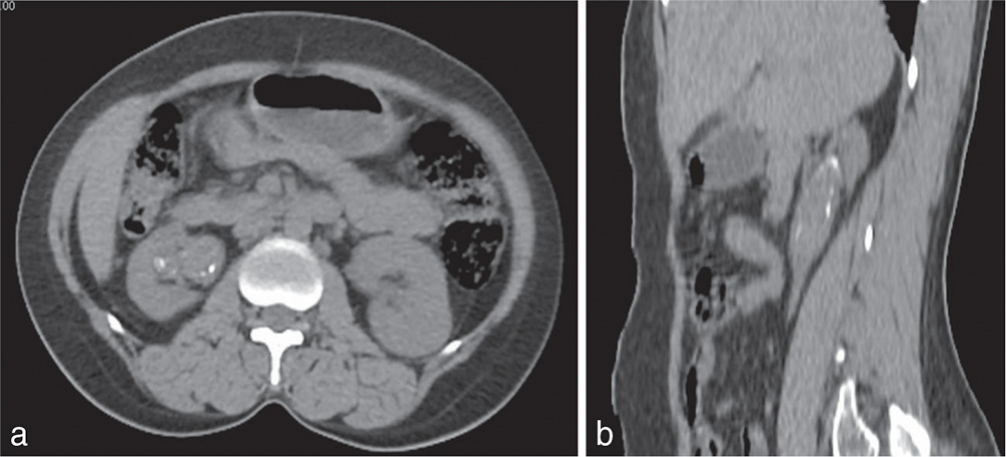
Plain CT scan axial (a) and sagittal (b) reconstruction showing an isodense mass lesion with a peripheral rim of calcification in the dilated renal pelvis.

**Figure 3.**
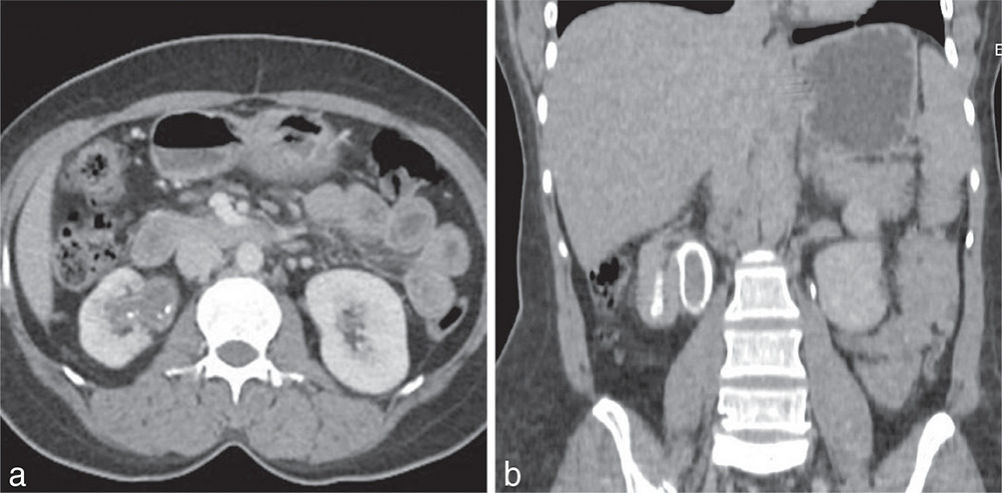
Contrast-enhanced CT images. (a) Nephrogram phase shows no enhancement of the lesion; subtle wall enhancement of the ureter is seen. (b) Delayed coronal reconstructed image shows contrast-filled urine all around the lesion.

Plain CT imaging of the abdomen (Figure 2a,b) confirmed the presence of right renal pelvic dilatation with an isodense intraluminal lesion in the renal pelvis with tiny peripheral calcifications. On contrast-enhanced CT scan (Figure 3a), a thin rim of ureteric wall enhancement was seen. No enhancement of the intraluminal lesion was seen. Coronal contrast-enhanced CT image (Figure 3b) showed contrast outlining of the lesion, with no attachment of the lesion to the renal pelvic wall.

**Figure 4.**
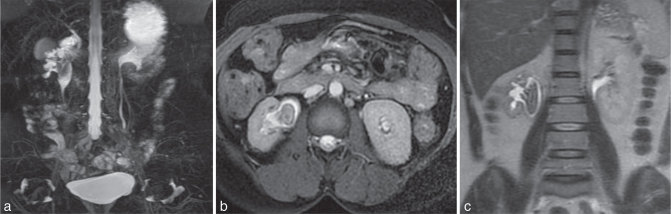
MR urogram (a) shows signal void within the dilated renal pelvis and axial *T*_2_ sequence (b) shows a hypointense lesion in the renal pelvis surrounded by hyperintense urine. Coronal *T*_2_ sequence (c) showing the contracted right kidney with the lesion in the renal pelvis mimicking a staghorn calculus.

MRI urogram (Figure 4a) showed a ballooned out renal pelvis with filling defect mimicking a calculus or mass and normal calibre ureter below. In axial short tau inversion-recovery sequence (Figure 4b), the lesion in the renal pelvis appears isointense with a peripheral rim of hypointensity that was surrounded by a rim of urine (hyperintense line all around the lesion). The renal pelvic wall is seen separately as an outer isointense margin. Coronal *T*_2_ sequence (Figure 4c) confirms that the renal pelvic wall is seen separate from the lesion. The right kidney appears smaller in size with moderately preserved cortex. The upper margin of the lesion appears to be a calyceal cast mimicking a staghorn calculus. The diagnosis was that of a right renal pelvic fungal ball causing obstruction and hydronephrosis.

### Treatment

**Figure 5.**
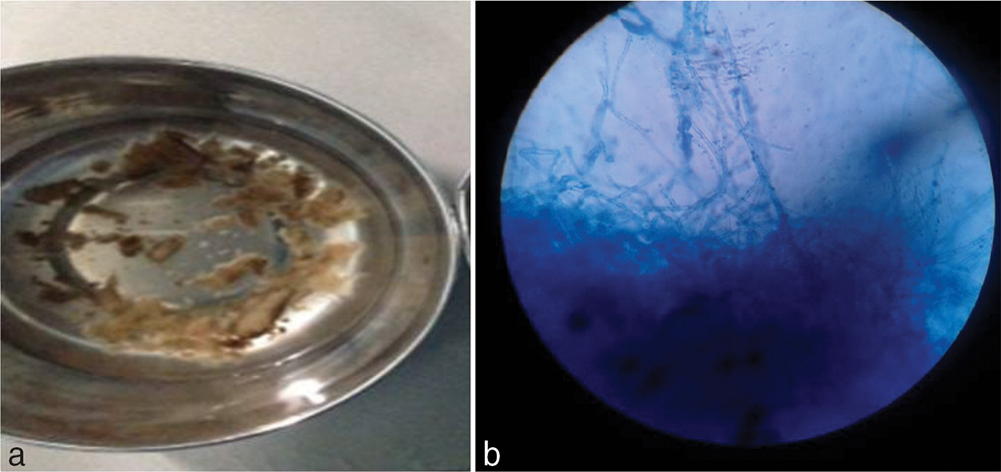
Post-endourological procedure specimen (a) showing bits of extracted tissues; microscopic picture (b) showing large pseudomycelia of coelomyecetes.

Endourologically extracted bits of tissue (Figure 5a) sent for culture and microscopy (Figure 5b) showed hyphae of coelomyecetes. The treatment was avoidance or control of risk factors, combination of endourological procedures and medical therapy. Our patient was initially started on oral fluconazole 200 mg once daily and injection of amphotericin was added at a dose of 0.5 mg kg^−1^. The patient underwent ureteroscopic retrieval of the fungal ball followed by instillation of amphotericin in the renal pelvis.

### Outcome and follow-up

The patient recovered well. USG at 6-month follow-up did not show any hydronephrosis or lesion in the renal pelvis.

## Clinical presentation—case 2

A 45-year-old male patient with diabetes on insulin was admitted with complaints of right loin pain and pneumaturia for 1 week. He had a history of ulcerative colitis that was diagnosed 15 years ago and was on treatment. He also had a history of right pelviureteric junctional stricture and pyeloplasty carried out. The patient was clinically stable and non-toxic at admission. Clinical examination showed a ballotable right kidney and tenderness over the right lumbar region. Routine urine examination showed fungal hyphae.

### Differential diagnosis

A provisional diagnosis of pyelonephritis with fungal infection was made clinically and the patient was referred for cross-sectional imaging.

### Imaging findings

**Figure 6.**
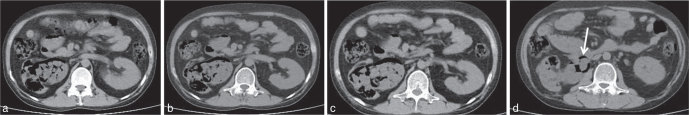
(a–c) Plain CT axial section showing multiple well-defined isodense oval lesions within the renal pelvis interspersed with air pockets and significantly thinned out renal parenchyma. (d) One of the lesions projects from the anterior wall (arrow).

USG of the abdomen showed poor visualization of the kidney owing to the presence of a significant amount of air in the renal parenchyma. Plain CT scan of the abdomen (Figure 6a–d) showed significant air collection in the renal collecting system. The parenchyma was thinned out. The renal pelvis was dilated. Multiple isodense oval lesions of varying sizes were seen in the renal pelvis outlined well by crescents of air. One of those lesions was seen projecting from the anterior non-dependent wall (arrow in Figure 6d). Contrast CT scan was not performed because of mildly elevated renal parameters. A diagnosis of EPN (Type 1 Huang and Tseng classification) with multiple fungal balls was made.

### Treatment, outcome and follow-up

**Figure 7.**
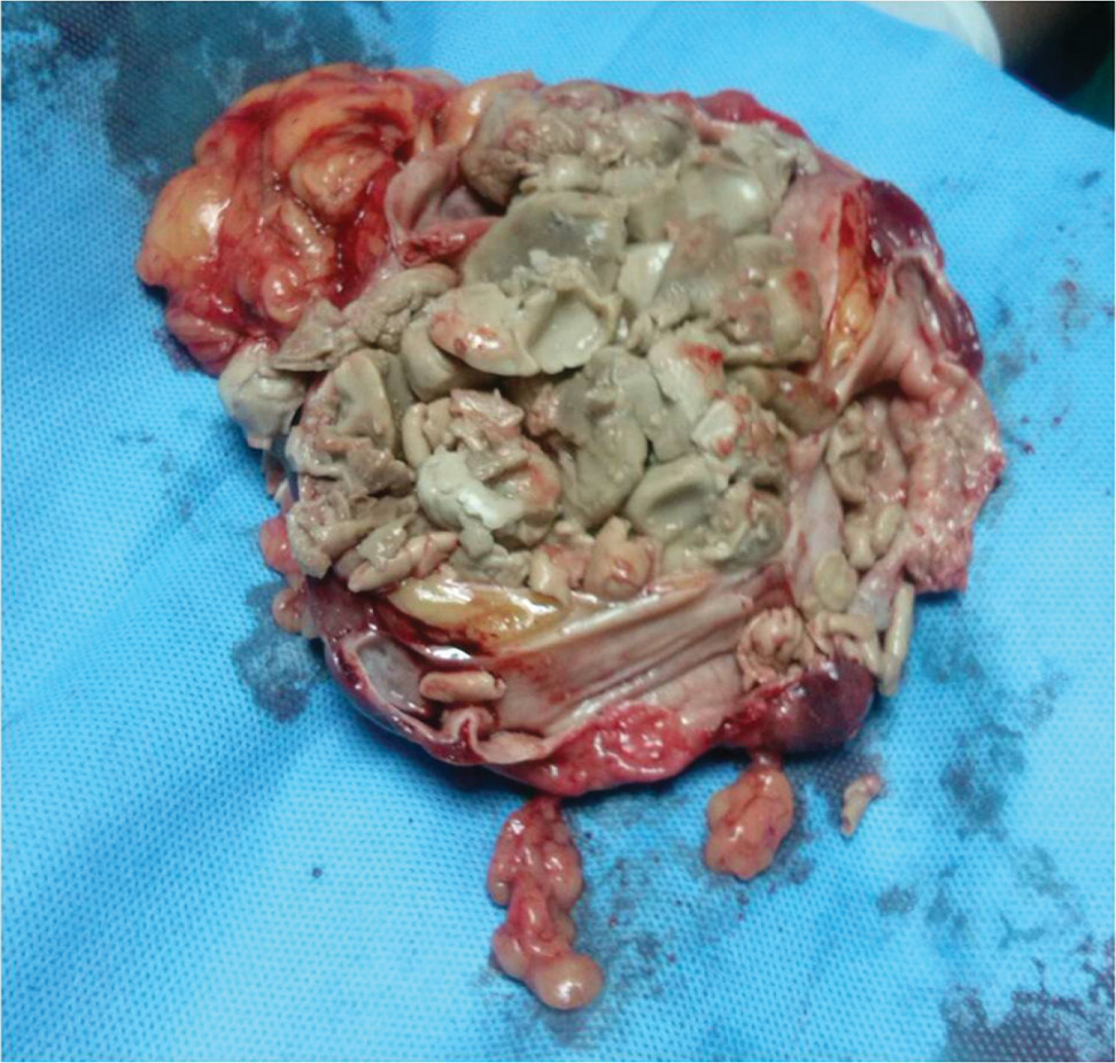
Nephrectomy specimen cut open showing thinned out renal cortex and renal pelvis completely studded with fungal ball.

The patient was started on oral flucanazole. Elective right nephrectomy was performed because of associated multiple high risk factors and thinned out renal parenchyma. The cut-open nephrectomy specimen showed multiple fungal balls (Figure 7). Specimen culture showed *C. albicans*. There were no post-operative complications and the patient remained well with normal renal parameters.

## Discussion

Upper urinary tract fungal infections are relatively uncommon, and fungal bezoar formation is particularly rare.^1,2^ Fungal bezoars are formed by localized collections or clusters of pseudomycelia, which may become quite large. It is more common in immunosuppressed individuals.^2^

Literature review reveals that renal involvement is commonly caused by disseminated candidiasis, but a primary infection can be caused by an ascending process or *via* urinary catheter drainage.^3^ It can affect the renal parenchyma or the drainage system and cause a cortical abscess or an obstructive intrarenal mass, commonly in the pelviureteric junction.^4^ It can present as renal colic, fever, dysuria, chills, vomiting or decreased urine output, and the age at presentation can vary from newborn to elderly.^2^

Majority of the cases described in the literature suggests that diabetes, immunosuppressed state, bladder catheterization and prolonged antibiotic use are a few of the high risk factors for opportunistic infection by fungus. *Aspergillus, Mucormycetes, Cryptococcus* and *Histoplasma* are other organisms known to infect the urinary system.^5^ A case of renal fungal ball with *Geotrichum candidum* has been reported in a post-partum patient.^6^ *G. candidum* infection has been reported commonly in renal transplant recipients.

To our knowledge, this is the first case of renal coelomyecetes fungal bezoar reported in the world. Coelomyecetes are a rare group of fungi, described more commonly in neonates and rarely in adults. Coelomycetes bear conidia within the structure; they are asexual fungi. There is no airborne spread. It is acquired only through implantation. Common diseases caused by coelomyecetes in humans have been reported in the eyes and skin.^7^

In our first case, a positive history of calculus disease treated with extracorporeal shock wave lithotripsy and double J stenting suggests possible implantation. While immunocompetent individuals resist infection, this patient with a history of diabetes mellitus and treatment for carcinoma of the breast had possible immunocompromise.

EPN is a life-threatening necrotizing infection of the kidneys with presence of gas in the collecting system, renal parenchyma or in the perinephric region, depending upon the severity. It is caused by bacterial infections such as *Escherichia coli, Pseudomonas, Proteus, Klebsiella, Clostridia* or by fungal infections such as *Candida tropicalis, C. albicans* and *Cryptococcus* and *Aspergillus fumigatus*.^8^ EPN is commonly seen in patients with diabetes and associated urinary tract obstruction. Both risk factors were present in our second case.

85% of cases are seen in females owing to the high incidence of urinary tract infection in women. CT scan is the diagnostic modality to confirm and know the extent of the EPN and status of obstruction. The treatment can be medical/surgical or combined, depending upon the risk factors. Low mortality with CT-guided percutaneous drainage with antibiotic/antifungal agents and supportive care has been reported by Chen et al,^9,10^ Tilden et al^11^ and many others. Early diagnosis of EPN is important because the patient can progress to septic shock rapidly.

The differential diagnosis for a renal pelvic intraluminal lesion is mass lesion, blood clot, debris and calculus.^2,3^ The mass usually shows enhancement and attachment to the wall of the pelvis. Blood clots appear hyperdense on the CT scan and blooming can be seen on the MRI gradient echo sequence. The debris can show a change in shape and a calculus appears hyperdense on the CT scan, with signal void on MRI. They can be differentiated with imaging. Specimen pathology will confirm the diagnosis.

## Learning points

1. Literature review reveals that, on USG, a fungal ball is seen as a mobile echogenic mass with or without shadowing or as a hypoechoic lesion with a rim of peripheral echogenic areas, as in our case.
2. On CT scan, isodense, oval, well-defined lesions, single or multiple, in the renal pelvis are seen; no attachment of the lesion to the wall of the ureter is seen on delayed contrast CT scan as contrast (urine) outlining the lesion.
3. The presence of tiny peripheral calcifications (areas of high attenuation) can be owing to encrustations.
4. On MRI, the lesion appears isointense, surrounded by a rim of urine (hyperintense line all around the lesion in *T*_2_ or short tau inversion-recovery sequence). The renal pelvic wall is seen separately as an outer isointense margin separate from the lesion.

With the increasing number of patients with immunosuppression, diabetes and posturinary procedure follow-ups, renal fungal bezoar should be considered as one of the differentials. While knowing the imaging characteristics helps in timely identification, confirmation helps in starting correct treatment.

## Consent

Written informed consent was obtained from the patient for publication of this case report, including accompanying images.
